# Anaplastic Thyroid Carcinoma With Predominant Intrathoracic Progression: A Case Report

**DOI:** 10.1155/carm/2164998

**Published:** 2026-04-27

**Authors:** Soichiro Ueda, Jin Kagatani, Yuriko Nakamura, Yu Asami, Shingo Tsuruoka, Natsumi Yazaki, Mamoru Sasaki

**Affiliations:** ^1^ Department of Respiratory Medicine, Japan Community Health Care Organization (JCHO), Saitama Medical Center, Saitama, Japan, jcho.go.jp; ^2^ Internal Medicine, Japan Community Health Care Organization (JCHO), Saitama Medical Center, Saitama, Japan, jcho.go.jp; ^3^ Department of Diagnostic Pathology, Japan Community Health Care Organization (JCHO), Saitama Medical Center, Saitama, Japan, jcho.go.jp

## Abstract

While most thyroid cancers have a favorable prognosis, anaplastic thyroid carcinoma (ATC) is highly aggressive and results in a poor prognosis. ATC frequently arises from preexisting benign goiters or highly differentiated thyroid cancers, typically presenting with rapidly progressing local symptoms and often accompanied by distant metastasis. We report a case of ATC that rapidly progressed to intrapulmonary metastases and malignant pleural effusion. Despite a previous histological diagnosis of benign goiter and the absence of local symptoms at the final presentation, the patient died just 20 days after the onset of symptoms. In patients with thyroid tumors accompanied by rapid progression of intrathoracic lesions, ATC must be considered as a differential diagnosis, even if they were previously diagnosed with a benign goiter.

## 1. Introduction

Thyroid cancer is a malignant disease with a good prognosis. Anaplastic thyroid carcinoma (ATC) accounts for only 1%–2% of thyroid cancers [1]. However, ATC has the worst prognosis among all solid tumors, with a 1‐year survival rate of 21.1% [2]. Although distant metastases are often present in the early stages of the disease, the most common presentation is rapid progression of local symptoms in the neck.

Here, we report a case of ATC with multiple intrapulmonary metastases and bilateral malignant pleural effusion (MPE) that progressed rapidly and became fatal within a short period. This case is thought to represent the development of ATC from a preexisting goiter, but no progression of local symptoms was observed until the end.

## 2. Case Presentation

A 43‐year‐old man was referred to our hospital because of a cough and chest pain on the left side for 7 days. Seven years ago, the patient was histologically diagnosed with benign goiter at a specialized hospital and was recommended for follow‐up, but the visit to the hospital was interrupted. The patient had no history of smoking or alcohol consumption. His vital signs were as follows: blood pressure, 121/78 mmHg; heart rate, 105 beats/min; respiratory rate, 20 breaths/min; and oxygen saturation, 100%. The body temperature was 37.4°C.

Auscultation of the lungs and heart revealed normal findings. He had long been aware of a right thyroid mass, but its size had not changed over the last 7 years or showed slight subjective shrinkage. The patient did not have any hoarseness or neck pain.

Laboratory investigations revealed a white blood cell count of 11.6 × 10^9^/L (normal range: 3.5–8.5 × 10^9^/L), hemoglobin level of 130 g/L (135–170 g/L), platelet count of 470 × 10^9^/L (150–350 × 10^9^/L), C‐reactive protein (CRP) level of 176.4 mg/L (0.00–1.40 mg/L), and procalcitonin concentration of 50 ng/L (< 500 ng/L). Thyroid function was normal, and serum thyroglobulin level was 34.4 μg/L (< 35.1 μg/L).

**FIGURE 1:**
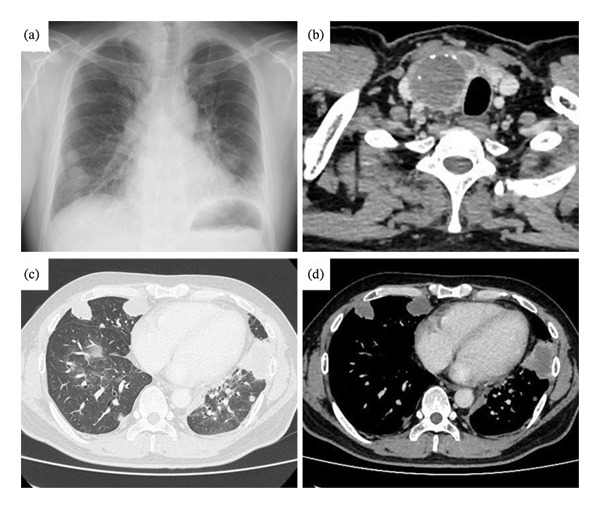
Chest imaging. (a) Chest X‐ray revealed multiple nodular shadows in both lower lungs. (b) Enhanced CT showed a 5‐cm tumor with poor contrast effect in the right lobe of the thyroid gland, compressing the right internal carotid artery and main bronchus. The tumor was accompanied by internal calcification and extensive contact with the capsular surface. (c) CT showed multiple mass lesions predominantly in both lower lungs. (d) Each lesion was accompanied by necrosis, as were the thyroid tumors.

Chest radiography revealed multiple nodules in both lungs (Figure 1(a)). Enhanced computed tomography (CT) revealed a 5‐cm tumor in the right lobe of the thyroid gland (Figure 1(b)), mediastinal lymphadenopathy, and multiple pulmonary nodules (Figure 1(c)). All lesions were accompanied by necrosis (Figures 1(b) and 1(d)). The tumor compressed the main bronchus and right internal carotid artery, and thyroid capsule infiltration was suspected. However, there was no evidence of invasion into the surrounding organs. Enhanced CT of the head was also performed, but no distant metastases were detected outside the lungs.

On the same day, he was admitted to the hospital for a full examination and diagnosis. Based on the imaging findings and clinical course, we considered the following as differential diagnoses: pulmonary metastases of thyroid cancer or lung cancer and septic embolism. The patient was administered antibiotics during the thorough examination.

**FIGURE 2:**
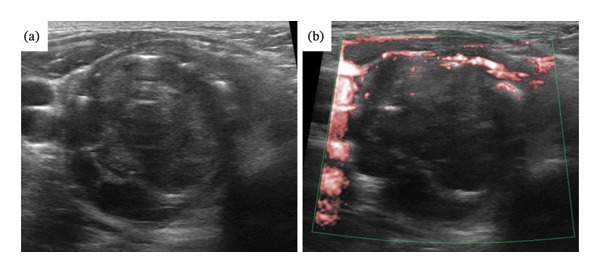
Ultrasonography showed a 4.5‐cm tumor in the right lobe of the thyroid gland. (a) The internal echoes were heterogenous. Lymph node metastasis was observed below the thyroid tumor. (b) The blood flow signals inside the tumor were poor.

Ultrasonography showed a 4.5‐cm tumor in the right lobe of the thyroid gland, with heterogeneous internal echoes and poor blood flow (Figures 2(a) and 2(b)), suggesting poorly differentiated carcinoma. Ultrasonography performed 7 years prior showed a 5.2‐cm mass and findings of goiter with benign follicular epithelium.

**FIGURE 3:**
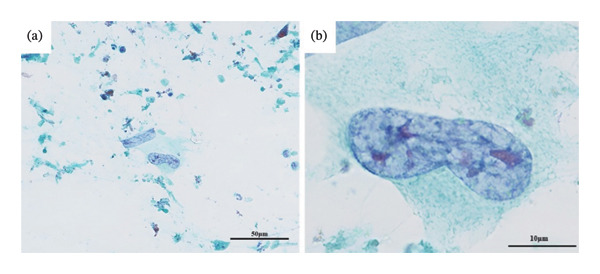
FNA from the right lobe of thyroid mass. (a) A few tumor cells were observed in the background of necrotic material (Papanicolaou stain). (b) The tumor cell showed large atypical cells with nuclear enlargement. Some multinucleated and spindle‐shaped cells with distinct nucleoli were present (Papanicolaou stain).

Fine needle aspiration (FNA) cytology of the right thyroid gland revealed large cells with background necrosis and enlarged nuclei, which were considered malignant cells (Figures 3(a) and 3(b)). These findings were not in line with those of follicular or papillary carcinoma, and poorly differentiated carcinoma or anaplastic carcinoma was suspected. Cytology of the same site, performed by a previous physician, revealed benign atypical follicular epithelial cells. The course of the disease during this period was unknown; however, transformation to malignancy was thought to have occurred.

**FIGURE 4:**
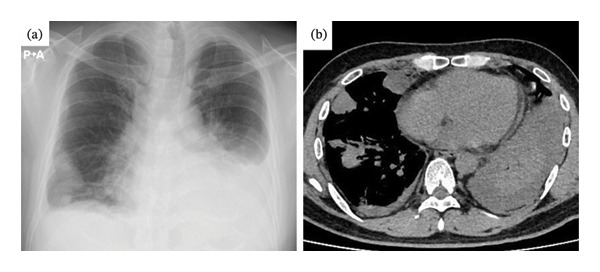
Chest imaging after disease progression. (a) X‐rays showed bilateral pleural effusions predominantly on the left side and increasing pulmonary nodular shadows. (b) CT revealed that each lesion was enlarged and the left lower lobe was collapsed due to pleural effusion.

Although biopsies of the pulmonary nodule or mediastinal lymph node specimens were also considered, the patient’s general condition deteriorated rapidly after admission, similar to respiratory failure due to progressive lung lesions. Moreover, chest pain on the left side gradually worsened, rendering bronchoscopy impossible. On the 6th day, left‐predominant bilateral pleural effusion was observed (Figures 4(a) and 4(b)).

**FIGURE 5:**
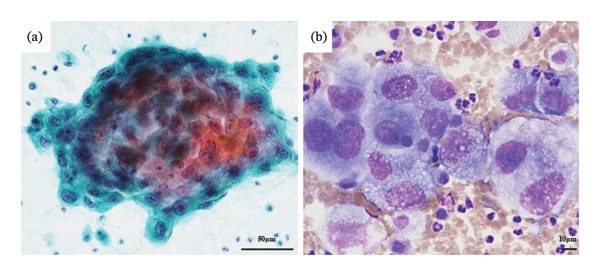
Cytology of pleural effusion. (a) Numerous clustered large cells with an unevenly distributed nucleus were seen in a neutrophilic, inflammatory background (Papanicolaou stain). (b) The cytoplasm of individual cells was somewhat pale and vesicular. The nucleus was round, with irregular size and shape, and some karyotypes were irregular. Multinucleated cells were also scattered (May–Giemsa stain).

The pleural fluid was bloody and exudative, and cytological examination revealed malignant cells, suggesting poorly differentiated or undifferentiated carcinoma (Figures 5(a) and 5(b)).

A pleural fluid cell block was prepared, and immunohistochemistry findings were positive for CK7 and negative for CK20 and Thyroid transcription factor‐1 (TTF‐1). Together with other immunostaining results, the primary site could not be identified. However, the staining pattern was consistent with ATC, and together with the FNA findings of the right thyroid gland, it was thought to be an MPE due to ATC. The CRP levels did not improve with antimicrobial therapy and remained elevated at 370.0 mg/L after admission. Blood and sputum cultures were also negative, and CRP was considered to reflect the severity of ATC disease.

The patient was diagnosed with multiple longitudinal lymph nodes, lung metastasis, and bilateral pleural effusion of ATC T3bN1M1 Stage IVC and was transferred to a higher medical institution on the 8th day after admission. However, he developed paralysis of the right upper and lower extremities during the transfer and was newly diagnosed with Trousseau’s syndrome. His general condition deteriorated, and he died shortly thereafter. The clinical course of the disease was rapid, lasting only 20 days from the onset of the first symptoms to death.

## 3. Discussion

In this report, we describe a case of ATC that rapidly progressed to intrathoracic metastases and became fatal within a short period.

Thyroid tumors are most often discovered incidentally during medical examinations or imaging tests, and the likelihood of malignant disease is low. In addition, most thyroid cancers are papillary carcinomas, which generally progress slowly and have a 5‐year survival rate of approximately 98.5% [3]. ATC is a rare form of thyroid cancer that is considered to be infrequent, accounting for 1%‐2% of all thyroid cancers [1]. Goiter is considered to be often preceded by highly differentiated thyroid cancers such as papillary or follicular carcinoma, which suddenly change in nature and transform into ATC; however, the mechanism of pathogenesis remains largely unknown. In contrast to other types of thyroid cancers, ATC has the worst prognosis, with a 1‐year survival rate of 21.1% [2]. The prognosis is even worse in cases in which surgery or radiation cannot be performed. In patients with distant metastases, as in this case, the 1‐year survival rate is only 14% [2].

While thyroid cancer generally exhibits a marked female predominance with a male‐to‐female ratio of approximately 1:3, ATC shows a relatively higher proportion of male patients. Furthermore, ATC is characterized by a significantly later onset compared to other histological subtypes, with a median age of 70 ± 12 years [4]. The onset of ATC in a patient in their early 40s, as presented in this case, represents an exceedingly rare occurrence, given the disease’s predilection for the elderly. Local symptoms, such as neck discomfort, pain, dysphagia, and dysphonia, often progress rapidly and trigger detection. Patients are often aware of a cervical mass, and the average tumor size at the time of initial diagnosis is 6.1 cm [4].

From its initial clinical onset, ATC typically exhibits extensive invasion into peritumoral tissues, including the surrounding muscles, trachea, and esophagus. Therefore, regardless of the tumor size or nodal status, all ATC patients are clinically staged as Stage IV. Similarly, distant metastases are often present at the time of the initial diagnosis, with the incidence rate estimated at 30% [4], with lung metastasis being the most frequent.

Blood test results often show elevated WBC counts and CRP levels, reflecting the progression of the disease. Thyroid function test (T3, T4, free T3, and free T4) and levels of antithyroid peroxidase (TPO) antibody and antithyroglobulin antibody may show transient abnormalities.

The recent American Thyroid Association (ATA) guidelines recommend molecular testing for patients with unresectable Stage IVB and Stage IVC ATC. The *BRAF* ^*V600E*^ mutation is frequent and is the most important molecular factor found in 40%–70% of ATC cases [5]. In the Phase 2 basket trial (Rare Oncology Agnostic Research) of dabrafenib (BRAF kinase inhibitor) plus trametinib (MEK inhibitor), investigator‐assessed overall response rate was 56%, and the median PFS and OS were 6.7 months and 14.5 months, respectively [6]. Taxane‐based anticancer drugs are recommended as bridge therapies until the results of molecular testing are known.

In this case, distant metastases (multiple pulmonary metastases and bilateral pleural effusions) progressed rapidly; however, there was no local progression, which is common in patients with ATC. Lung cancer is the most common primary cause of MPE, followed by breast cancer, malignant lymphoma, and ovarian cancer. MPEs caused by thyroid cancer are rare. ATC is often accompanied by distant metastases and is considered a possible cause of MPE. In many ATC cases, pulmonary metastases and MPE appear and worsen following local disease progression. However, there are only a few detailed reports of ATC with MPE confirmed via pleural fluid cytology. There was only one case in which malignant cells were found in pleural effusion without any preceding diagnosis of thyroid cancer, such as papillary carcinoma. Sodhi et al. reported a case of ATC that led to the diagnosis of bilateral pleural effusion and showed malignant cells arranged in three‐dimensional ball clusters and groups on pleural fluid cytology [7]. The cytological findings were similar to those in our case. In their report, bilateral pleural effusions were progressive; however, the thyroid tumor was not large (approximately 2 cm) and lacked local symptoms, as in our case. Based on imaging findings at the time of the initial diagnosis, multiple intrapulmonary metastases of thyroid cancer were one of the differential diagnoses, and the possibility of transformation to ATC was also considered. However, this case was characterized by an uncommonly young age for ATC and an atypically stable clinical presentation; the thyroid mass had shown no growth for 7 years following a benign cytology result. These factors confounded the prediction of the subsequent rapid clinical deterioration. The disease followed a remarkably fulminant progression, leading to the patient’s death just 20 days after symptomatic onset. Even with proper diagnosis and management, it would have been difficult to save the patient’s life. The only possible treatment would have been to perform a *BRAF* ^*V600E*^ test with core needle biopsy when ATC was suspected using FNA of the thyroid gland and to use chemotherapy, such as paclitaxel, as a bridging therapy before the results were known.

This case indicated that ATC has a poor prognosis and that the rapid progression of distant metastases is fatal, even if local symptoms are scarce. When investigating the cause of multiple pulmonary metastases or MPE, we tend to exclude the possibility that a tumor previously found to be histologically benign may actually be the cause. However, ATC can transform from a benign goiter over a long period and become fatal within a short period, as indicated in this report. When investigating the primary site of intrathoracic lesions in cases of thyroid tumors, ATC must be considered as a differential diagnosis, regardless of the previous diagnosis.

Finally, regular clinical surveillance, including physical examination, ultrasonography, and thyroid function testing every 6 to 12 months, is essential for patients with benign thyroid nodules. Should any rapid growth, suspicious ultrasonographic changes, or new‐onset symptoms occur, prompt FNA is mandatory to exclude the possibility of malignancy.

## 4. Conclusions

ATC is a malignancy with a poor prognosis and can be fatal within a short period. Therefore, prompt diagnosis and treatment are essential. A history of benign goiter does not exclude the development of ATC. Clinicians must consider the possibility of ATC in patients with thyroid tumors who present with rapidly progressing intrathoracic metastases, even in the absence of worsening local symptoms.

## Funding

The authors have nothing to report.

## Consent

A written informed consent was appropriately obtained from patient’s family for the publication of this case report.

## Conflicts of Interest

The authors declare no conflicts of interest.

## Data Availability

The data of this case report are available from the corresponding author upon reasonable request.
